# Different approaches for interpretation and reporting of immunohistochemistry analysis results in the bone tissue – a review

**DOI:** 10.1186/s13000-014-0221-9

**Published:** 2014-11-29

**Authors:** Nickolay Fedchenko, Janin Reifenrath

**Affiliations:** Small Animal Clinic, University of Veterinary Medicine, Foundation, Bünteweg 9, 30559 Hannover, Germany; Department of Pathological Anatomy and Forensic Medicine, SE “Dnipropetrovsk Medical Academy of Health Ministry of Ukraine”, Dzerginskogo st. 9, 49044 Dnipropetrovsk, Ukraine

**Keywords:** Immunohistochemistry, Score, Grading, Semiquantitative, Bone

## Abstract

**Background:**

Immunohistochemistry (IHC) is a well-established, widely accepted method in both clinical and experimental parts of medical science. It allows receiving valuable information about any process in any tissue, and especially in bone. Each year the amount of data, received by IHC, grows in geometric progression. But the lack of standardization, especially on the post-analytical stage (interpreting and reporting of results), makes the comparison of the results of different studies impossible.

**Methods:**

Comprehensive PubMED literature search with a combination of search words “immunohistochemistry” and “scoring system” was performed and 773 articles describing IHC results were identified. After further manual analysis 120 articles were selected for detailed evaluation of used approaches.

**Results:**

Six major approaches to the interpretation and presentation of IHC analysis results were identified, analyzed and described.

**Conclusions:**

The overview of the existing approaches in evaluation and interpretation of IHC data, which are provided in the article, can be used in bone tissue research and for either better understanding of existing scoring systems or developing a new one. Standard multiparametric, semiquantitative IHC scoring systems should simplify and clarify the process of interpretation and reporting of received data.

**Virtual slides:**

The virtual slide(s) for this article can be found here: http://www.diagnosticpathology.diagnomx.eu/vs/13000_2014_221

## Introduction

The main aim of any histopathological investigation is the identification of a pathological process, therefore special diagnostic features are necessary. Revealing of such features in bone tissue is concerned with several differences compared to other tissues. They start from the very beginning of the long chain of bone specimens obtaining and preparation: bone tissue needs prolonged fixation, often decalcification, special media infiltration and embedding, special equipment for cutting of the tissue specimens (heavy-duty microtomes, diamond circular or wire saws), and even grinding machines for section thinning and grinding [1,2].

Unfortunately there is still no staining procedure invented, which is able to obtain specific information about all desired structures, such as osteoid, mineralized bone matrix, glycosaminoglycans and many others on one slide. To receive important information scientists choose the relevant staining method from a wide range of available ones nowadays. Many excellent reviews presented a variety of staining methods, and their pros and cons [1,3–6]. Among of all methods, immunohistochemistry is a well-established tool, which is widely used to help identifying a wide spectrum of specific pathological processes and which is used in experimental research involving bone tissue. Besides descriptive analyses, multiparametric, semiquantitative scoring systems for evaluating different bone parameters represent an universal approach to include histopathologic information in biomedical research [7–9].

In general, one of the most important attribute of any scientific research is its language or nomenclature. The first widely accepted standardization of bone tissue nomenclature was made by Michael Parfitt in 1987 [10]. It was widely accepted and improved markedly the ability of bone researchers to communicate with each other and with nonspecialists, leading to a broader understanding and appreciation of bone research data. After 25 years these recommendations were revised and published in 2012 by David W Dempster and his coauthors [11].

Contrary to the general bone tissue nomenclature, there is still a huge gap in the standardization of IHC methods. IHC is a multistep procedure, and each step is vital. The importance of using standardized rules and environment on each stage of the method is stated in various articles and monographs [12–25]. According to existing conceptions, all variables implying on IHC methods are divided into 3 groups:

1. Pre-analytical variables of IHC tests include Any and all steps in tissue processing, starting from tissue sample obtaining (prolonged ischemia, delayed fixation, etc.), type and length of fixation, decalcification, and elements of tissue handling (proper specimens orientation, careful notation of surgical margins, slicing into sections at 2 to 5 mm intervals, adequate naming, etc.). Unfortunately, pre-analytical variables cannot be controlled closely, unless you perform all the stages by your own or in certified laboratory [12,15,17,18,21–23].
2. Analytical variables of IHC tests include slide thickness, choosing of antibody clones and their titration, choosing the detection systems and, of course, antigen retrieval (AR) procedure. Current IHC detection systems include peroxisae-anti-peroxidase, the avidin-biotin complex, the biotin-streptavidin amplified systems, tyramine amplification method, immuno–rolling circle amplification, and the polymer enzyme system [26,27]. Antigen retrieval procedures include enzymatic digestion, acid treatment, alkaline hydrolysis, detergent treatment, using the urea solution, refixation with Zn-solution, freeze and thawing, freeze and drying, and of course heating [14,27–29]. AR method should be carefully selected, because many antigens are very sensitive for selected approach, and AR may either enhance the result or completely destroy the target substance of interest [30].
3. Post-analytical variables of IHC tests include interpretation and reporting of the results [31]. Despite all existing recommendations, post-analytical variables are the most frivolous part of many experiments using IHC diagnostics. Misinterpretation of positive and negative results, inappropriate morphological context, unclear scoring systems, and inadequate statistical analysis make it impossible to perceive any data and compare it to other scientific information.

The last step of IHC variables and particularly the scoring systems are the main topic of this review. The fundamental characteristics of a scoring system were suggested by Crissman et al., and included the following: (1) scoring system should be definable, (2) it should be reproducible, and (3) it should produce meaningful results [32]. Gibson-Corley et al. also described some key principles for an appropriate scoring system and data evaluation [33]:

- *“Masking”* of the experimental material to reduce the subjectivity of valued scores;
- a thorough *“Examination”* of all tissues/slides with creation of a context for scoring tissue lesions;
- specifying *“Lesion parameters”*, which then could be used as score categories;
- using a clear *“Scoring definitions”* will improve understanding of presented data and increase repeatability of scoring system;
- whenever possible, use *“Interpretation Consistency”* which imply that all the samples are scored by the same scientist in a reasonable period of time.

Semiquantitative scoring systems are widely used to convert subjective perception of IHC-marker expression by histopathologists into quantitative data, which is then used for statistical analyses and establishing of the conclusions. Without scoring system the description of received data can be provided only with subjective perception, expressed in such adjectives as “strong”, “weak”, “absent” with modifiers as “more” or “less”, like Sojo et al. in evaluation of VEGF and BMP-2, −4 on lengthened rat femurs [34]. Of course, this approach is used by each pathologist while examining the slides, but without conversion into a scoring system – they are just subjective expressions of assessments of solely one pathologist. To reduce subjectivity it is recommended to have at least more than one observer in the study [35].

Most semiquantitative scoring systems usually include multiple parameters which are separately quantified on an ordinal scale and finally combined in a total score. Average scores of the different experimental groups can then be compared by non-parametric statistical tests [7]. The selection of the parameters should be based on the scientific hypothesis or question together with the morphological features of expression of IHC markers which are used in an experiment. The “golden standard” in IHC scoring is defined for the evaluation of only 3 markers so far: Her2/neu, estrogen (ER), and progesterone (PR) for which testing guidelines have been developed [36].

For many IHC markers scientists design an individual scoring system, which might be the best possible way to answers the particular scientific question. Lack of standard scoring systems for most IHC markers and particularly for bone tissue leads to the impossibility of the comparison of the results with other studies [37,38].

The present review is aimed to provide the reader with an overview of the existing approaches in evaluation of IHC markers which can be used in bone tissue research and for either better understanding of existing scoring systems or developing a new one.

## Review

### Methods

**Figure 1:**
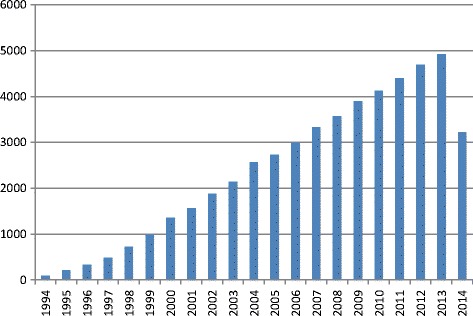
**Count of articles, mentioning “VEGF” from 1994 to 2014 according to PubMed.**

**Figure 2:**
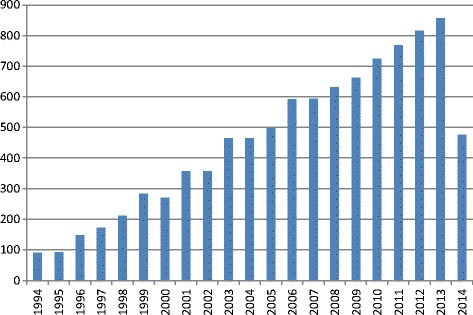
**Count of articles, mentioning “Bone Morphogenic Protein” from 1994 to 2014 according to PubMed.**

Inclusion criteria for comprehensive literature search were a description of IHC results with or without scoring system. The priority was given for the scoring systems for IHC markers that can be used in bone tissue studies. Among such markers were Vascular Endothelial Growth Factor (VEGF), Bone Morphogenic Proteins (BMP), Osteocalcin (OCN), Osteopontin (OPN), and some others with developed scoring systems. Exemplary for VEGF and BMP the tendency in ongoing immunohistochemistry researches is shown in Figure 1 – their number is growing in geometric progression. During last 20 years (from January 1994 to July 2014) the number of articles mentioning VEGF was more than 50000. Bone Morphogenic Protein was mentioned in 9530 articles (Figure 2).

Using a comprehensive PubMED search with a combination of search words “immunohistochemistry” and “scoring system” 773 articles were identified. After further manual analysis 120 articles were selected for detailed evaluation of used approaches for interpretation and reporting of immunohistochemistry analysis results.

### Results

**Figure 3:**
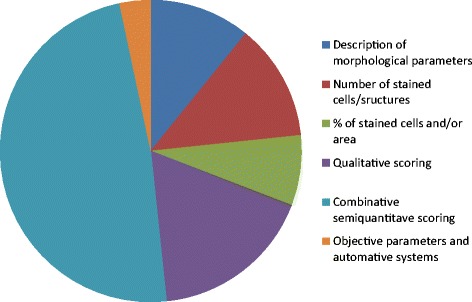
**Ratio of different IHC data interpretation and presentation methods in selected articles.**

A widely accepted scoring system for immunohistochemistry does not exist yet. The amount of IHC markers used in clinical and experimental research is constantly growing, and so do the amount of researches and data in the field. A closer manual analysis of the selected 120 articles allowed us to identify six major approaches to the interpretation and presentation of received results (Figure 3).

#### Description of morphological parameters

This approach is the example of pure qualitative analysis of received information. Verbal description works well if the amount of slides is small and no further statistical analysis of received information is planned; for example, for pilot studies or if IHC analysis is not the main method in an experiment. For presenting the data in the article authors use a literal description of a histological picture (which cells or tissue components were immunopositive) and properties of IHC expression (weak/moderate/strong intensity, staining pattern, background, etc.) [34,39–49]. Detailed examination and description of alkaline phosphatase (ALP), collagen type I (COL I), osteonectin (OTN), OPN, OCN, and bone sialoprotein (BSP) expression in cellular and matrix components of bone was performed by Knabe et al. [49].

Unfortunately, if the results are presented only in a descriptive form, they cannot be compared to other studies directly. However, sometimes such method gives very valuable details, which may be hidden by scoring system categorization [33,50].

#### Evaluation of number of IHC-positively stained cells and structures

This is quite simple and commonly used approach in evaluating IHC results. Authors count the absolute quantity of positively stained cells for each investigated IHC marker in different experimental groups [51–53]. For example, Ishihara et al. counted the number of BMP-2 stained cells in decalcified rabbit nasal bone [52].

IHC markers (factor VIII, CD31, CD34, CD105, VEGF and its receptors, etc.) are often used to establish microvessel density (MVD) [54–64]. This parameter is often presented as a number of microvessels per square millimeter or mean value with standard deviations. For including a microvessel into a count it should be presented as any brown-stained endothelial cell or endothelial-cell cluster that was clearly separate from adjacent microvessels, tumor cells, and other connective-tissue elements [65].

The main problem of cell and structures counting, that it must be very clearly mentioned which cells and/or structures were considered to be “positive”. If the IHC staining is not homogeneous, cell populations with different staining properties can be counted separately [66]. Sometimes background staining may lead to misinterpretation [25] and as for the bone tissue, the expression of many IHC markers is observed not only in the cells, but in the osteoid and bone matrix either [67,68].

Results in studies using this method in most cases are presented as mean values of positively stained cells (and/or structures) among counted experimental groups with their standard deviations [51–64]. If the IHC marker has a high affinity to cells, then the process of positive cells counting may be optimized by some special methods [69].

#### Evaluation of IHC-positively stained cells and/or area ratio

This approach seems to be more time consuming, therefore it is more informative. Researchers count the percentage of positive immunolabeled cells over the total cells in each selected area [70]. This method can be automated with the use of special plugins for computer counting of general amount of cells and positively stained cells [71].

Because slides are stained separately for each IHC marker (if otherwise is not stated), the % of positively stained cells is counted separately either. The relation of positively stained cells sometimes is presented in the labeling index (the ratio number of positively stained cells/total number of cells × 100) [72,73]. Wittenburg et al. evaluated for OCN, OTN, OPN, COL I, CD34, and CD68 the positively stained areas in relation to the total bone surface per section in percentage [2].

As in the second approach, where absolute quantity of cells was calculated, in scoring of % of immunopositive cells all criteria should be clearly mentioned: which cells and areas were considered to be “positive” or “negative” and why.

The measurement of both, percentage of positively stained cells and area, was performed by Ramazanoglu et al. in the investigation of COL I, BMP-2\4, OCN, and OPN [67]. In this study immunopositive cells were counted in each region of interest (ROI) using a counting grid and their proportion among the total counterstained cell population was analyzed. For COL I stained areas of the ROI were digitally marked and the percentage of stained areas was determined using a computer program.

Usually the combination of quantitative and qualitative parameters leads to expression of received data in a combined scoring systems, which are described later in this article. But the amount of positively stained cells and their relations can be expressed via a simple qualitative scoring system, when certain percentage is given a certain score value [74,75]. Such approach was performed by Sulzbacher et al.: “++” score was given for 50–95% of positive stained tumor cells; “+” score for 10–49% of tumor cells positive; “−“ score when less, than 10% of tumor cells or no visible staining was observed [76]. Semiquantitative scoring with numbers instead of “+” signs can be used either, like did DeRycke et al. in their evaluation of S100A1 expression in ovarian and endometrial endometrioid carcinomas [77]. In this case investigated slides were assigned a score of 0 (no staining), 1 (<10% of neoplastic cells staining), 2 (10%–50% of neoplastic cells staining), or 3 (>50% of neoplastic cells staining) [77,78].

Results in studies, measuring the relations of IHC-stained cells and areas, are presented as mean values for % of positively stained cells with their standard deviations [2,72–75,79,80].

#### Qualitative scoring

As already described in the first part of this article, qualitative interpretation of IHC data is commonly used among scientists. In addition to the description of the evaluated parameters scientists may use qualitative scoring systems to interpret received data, usually the force of IHC staining in different investigated areas. Score ranks usually lie in a range from “negative” (mostly marked as “-”) to “positive”, which may be signed with different amount of “+” depending on how many other categories lay between these border parameters [79,81–84]. Most common spectrum of categories, describing different force of IHC expression in investigated groups, include: “negative”(−), “weak”(+), “moderate”(++), “strong”(+++) and their variations [85–91]. If the categories are signed with a numeric value instead of signs, then this approach transforms from qualitative to semi-quantitative [16,20]. Osteoprotegerin (OPG), receptor activator of nuclear factor-k ligand (RANKL), ALP, OPN, VEGF, tartrate-resistant acid phosphatase (TRAP), COL I, and OCN were assessed using a semi-quantitative ranking that ranged from 0 for no labeling to 4 for intense labeling in the of onlay bone graft remodeling by Hawthrone et al. [92]. Same approach with some extension of scoring groups was used in evaluation of VEGF, BMP-2 and core-binding factor alpha 1 (CBFA1) by Guskuma et al. [93].

Another variant of data presentation is scoring the force of IHC expression among different cell populations and tissue components. An example of this method is demonstrated by Yu et al. for scoring immunoreactivity for BMPs, BMP antagonists, receptors, and effectors in different cell populations during nonstabilized fracture healing [94]. Similar method was used by Li et al. for reporting relative abundances of BMP-2 and other IHC markers in uterine structural components and cells [90,95] and by Koerdt et al. in the study of the role of oxidative and nitrosative stress in autogenous bone grafts to the mandible [96].

A more complicated method of assigning different criteria for staining intensity was used by Ding et al., which included assignment of the intensity of staining using a scale of 0–10 (with 0 indicating a lack of brown immunoreactivity and 10 reflecting intense dark brown staining) by three observers. All observers evaluated all slides and observations outside of the 5th to 95th percentile of the remaining observations were considered outlying data and were excluded from analysis. After that the mean was calculated and the results were converted into grades: 1–3 score was assigned “+”, 4–6 was “++”, more than 7 was “+++” [97].

If the results in reports are presented as graded on a scale from “ − ” to “ + … + ” they may look more demonstrative, but the range of statistical methods is limited without a conversion to a numeric ordinal score for corresponding staining intensity [98]. However, only two groups, showing “positive” and “negative” expression of IHC marker, may be already compared statistically [99].

#### Combinative semiquantitative scoring

The most universal way to create a scoring system is to combine all existing approaches into a new one. There are quite a lot of examples of combined multiparameter scoring systems and in this review we will focus on the most recent and widely used ones. In multiparameter scoring systems the semiquantitative approach is used: investigated parameters are valued points from 0 to 4, 6 or even 18 depending only on depth of categorization of the used scoring systems. A small number of score categories may reduce the sensitivity of the scoring system, but a large number of ordinal scores may cause difficulty in score assignment as the distinctions between categories become less obvious. This leads to a less repeatability of the scoring system with large number of categories. Some authors suggest that to maximize detection and repeatability of the scoring system, it should contain an average of four to five score levels [100,101].

Simple combinative scoring system for evaluation of OCN and OPN expression was used by Bondarenko et al. [68]. Combination of quantitative and qualitative criteria in the semiquantitative scoring system was used in the study of VEGF-A, VEGF-C and fibroblast growth factor 2 (FGF-2) by Torre et al. [102]. The authors combined cells percentage with a force of IHC-staining and assigned to each field a value from 0 to 4 (0, negative; 1, <5% of the cells with positive staining; 2, between 5 and 50% of the cells with positive staining; 3, more than 50% of the cells with weak staining and 4, more than 50% of the cells with strong staining). The characteristics of selected scoring systems are shown in the Table 1. Similar approach was demonstrated by Jin et al. for evaluation of BMP-2/4, −5 and BMP protein receptor, type IA, but they did not count the intensity of staining [103].

**Table 1:** **Examples of combinative scoring system for histomorphometry**

**Score**	Bondarenko et al. for OCN* **[** 68 **]**	**Bondarenko et al. for OPN** **[** 68 **]**	**Torre et al. for VEGF-A, VEGF-C and FGF-2** **[** 102 **]**
0	-	-	Negative
1	<25%	Expression in cells only	<5% of the cells with positive staining
2	25-50%	Expression in cells and osteoid	Between 5 and 50% of the cells with positive staining
3	50-75%	Focal expression in mature bone	>50% of the cells with weak staining
4	>75%	Diffuse expression in mature bone	> 50% of the cells with strong staining

*OCN expression was evaluated as a percentage rate of immunopositive peri-implant bone tissue to all peri-implant bone area.

There are a lot of different approaches in establishing the evaluation criteria and corresponding scoring points. They are closely connected to the scientific goal of the experiment and properties of used IHC markers. Most criteria include percentage of positively stained cells and intensity of observed staining [104]. Unfortunately it is not always clear, how authors manipulate with their scoring systems. For example, Megumi et al. scored the percentage of BMP-7-positive stained cells and the intensity of the staining, but it is not clear how the intensity (valued from 1+ to 3+) implied the percentage (also presented in score values ranging from 1+ to 3+) [105]. The scoring system is very important in further statistical analysis of received information, because it directly determines the variability of achieved results [100,106] and statistical validity directly depends on the variability of representation [107]. Sometimes authors can perform simple manipulations to extend the range of score values. For example, Klein et al. for VEGF scoring added proportion score values to staining intensity score and received a range of values points from 0 to 6 [108]. Two years later the same author increased the range of points from 6 to 9 by changing arithmetical operation from addition to multiplication (Table 2) [109]. Such manipulation increase the variation row, which gives more statistically reliable results [110].

**Table 2:** **Scoring system used by Klein et al**

**A % of IHC + labeled cells**	**B intensity of IHC reaction**	**Final score**
0 = 0%	0 = no reaction	A + B = range from 0 to 6 [108]
1 = <30%	1 = weak	
2 = 30-60%	2 = mild	A × B = range from 0 to 9 [109]
3 = >60%	3 = strong	

Three examples of widespread combined scoring systems are Allred-score [96], immunoreactive score (IRS) [111] and H-score [112], which are commonly used for IHC evaluation of progesterone and estrogen receptors. Although these receptors are not expressed in bone tissue, these scoring systems considered to be “gold standard” in IHC-data evaluation and presentation They are widely accepted and recommended by leading associations and organizations [22,36,113,114]. The Allred scoring system combines the percentage of positive cells and the intensity of the reaction product in most of the examined fields. The two scores are added together for a final score with eight possible values. Scores of 0 and 2 are considered negative. Scores of 3 to 8 are considered positive (Table 3) [115,116].

**Table 3:** **Allred scoring system**

**Proportion score A**	**Positive cells, %**	**Intensity**	**Intensity score B**
0	0	None	0
1	<1	Weak	1
2	1 to 10	Intermediate	2
3	11 to 33	Strong	3
4	34 to 66	**Final score range (A + B): 0-8**	
5	≥67		

A similar approach to Allred score is demonstrated in so-called “quickscore” system, with the differences in assigned values from 1 to 6 in proportion category A (1 = 0-4%, 2 = 5-19%, 3 = 20-39%, 4 = 40-59%, 5 = 60-79%, 6 = 80-100%), also multiplication is recommended instead of addition for processing of final score range [117]. In literature Allred score is used for BMP-6 [118,119] and OPN [120] expression evaluation. According to Kejner et al. they used this scoring system for BMP-6 evaluation, but after authors modifications the score range was reduced to 4 categories, which described only intensity of staining: 0 (Low), 1 (Mid-Low), 2 (High-Mid), or 3 (High), which is actually not an Allred score anymore [119].

The H-score is determined by adding the results of multiplication of the percentage of cells with staining intensity ordinal value (scored from 0 for “no signal” to 3 for “strong signal”) with 300 possible values. In this system, <1% positive cells is considered to be a negative result [112,121]. According to Dabbs et al., H-score has a broader dynamic range compared to Allred score [9].

The immunoreactive score (IRS) gives a range of 0–12 as a product of multiplication between positive cells proportion score (0–4) and staining intensity score (0–3) (Table 4) [111]. IRS was used for expression of wide spectrum of IHC markers (BMP and its receptors, VEGF, vWF and others) in bone studies by Koerdt et al. [122,123]. For evaluation of BMP-6 reaction the IRS score with some modifications was used by Raida et al., but in the example used by authors the calculation of IRS is performed by summarizing of different score values [124]. Even more controversial approach in calculation of IRS score we can observe in the evaluation of BMP-2 score by de Carvalho et al., where authors mentioned, that they scored percentage of positive cells, but there were only two categories of stain intensity: score 1 (absent or weak expression) and score 2 (strong expression); and it is unclear what further manipulations authors performed with the score values – addition or multiplication [125].

**Table 4:** **The immunoreactive score (IRS)**

**A (percentage of positive cells)**	**B (intensity of staining)**	**IRS score (multiplication of A and B)**
0 = no positive cells	0 = no color reaction	0-1 = negative
1 = <10% of positive cells	1 = mild reaction	2-3 = mild
2 = 10-50% positive cells	2 = moderate reaction	4-8 = moderate
3 = 51-80% positive cells	3 = intense reaction	9-12 = strongly positive
4 = >80% positive cells	**Final IRS score (A × B): 0-12**	

If the examined sample stains for IHC marker heterogeneously, then each intensity of staining is scored independently and the results are summed. The example of such approach is given by Kraewska et al.: when a specimen contained 50% of the tumor cells with moderate intensity (2 × 2 = 4), 25% of tumor cells with intense immunostaining (1 × 3 = 3), and 25% of cells with weak intensity (1 × 1 = 1), the score was 4 + 3 + 1 = 8 [126].

Allred score, “quickscore”, H-score, and IRS are aimed only to the cellular staining evaluation and without modifications cannot be used for expression of extracellular staining.

#### Evaluation of objective parameters and automated approaches for calculation and scoring

Calculation of objective parameters such as optical density of positively IHC stained areas is a very perspective field, because until today the most common approach for analysis and interpretation of the IHC staining is a time-consuming and subjective manual procedure [71]. Due to broad scoring categories, nonstandardized approaches, subjectivity and variability of purely visual inspection the method of manual scoring IHC slides is less than precise [127]. In this review we briefly discuss major aspects of evaluating and scoring of some IHC parameters, which can be used in bone tissue research. One of the parameters that can be obtained and measured after IHC of bone tissue is Integrated Optical Density (IOD). This parameter was evaluated by Dehao et al. for VEGF expression [128] and by other authors for different IHC markers [129,130]. Results in experiments, measuring objective parameters, are presented in mean values of calculated parameter with their standard deviations. Of course, measuring objective parameters significantly reduces amount of subjective judgment which may implement the results. But high consumption of observer’s time makes it almost impossible to use any manual scoring in a large screening application. In such cases using personal computers with special analytical software may be the only alternative. The automation has penetrated in almost all fields of IHC [131,132], but interpretation and analysis of results remain an unreached milestone. Some products for automated measure are already present on the market and used in different experiments [71,127,133–136]. Kraan et al. compared manual and automated measurements of IOD and number of immunopositive cells in their work [137].

Rizzardi et al. compared pathologists manual scoring system with digital image analysis systems using digital data based on IHC-positive area (%Pos) and data combining area and staining intensity (OD × %Pos) [78].

Unfortunately, available automated systems are too far from ideal: some programs are not able to isolate individual cells, but most are still not capable for interpretation of morphological features [127]. Another major disadvantage of such systems are the costs and special skills required for the introduction and maintenance of all system components (software, hardware) [137]. On the other hand, manual scoring is not suitable for a large massive of data analysis [138], and in the authors opinion, the improvement of automated image analysis systems is just a question of time.

## Conclusions

Summarized, the six listed approaches for evaluation, interpretation, and presentation of received experimental IHC information contain significantly different data. Which approach is chosen depends only on the researcher’s opinion. Selection of an existing or developing of a new scoring system should be performed as early as possible, probably on such stages as developing the experimental design, purposes formationing and choosing tissue sampling parameters.

This review gives an overview on currently available approaches for evaluating and presenting data of bone immunohistochemistry, which may also be used in any other IHC field. In the authors opinion, a good scoring system is one of the key factors for any experiment. It helps to connect a specific scientific question with a clear presentation of achieved results. Properly selected or even newly developed scoring system will significantly increase the scientists productivity, save time and money.

Individual scoring systems for particular IHC marker may be the best possible way to answer the special scientific question, but lack of standard scoring systems for most IHC markers, and particularly for bone tissue, leads to the impossibility of the comparison of the results with other studies. Developing of standard multiparametric, semiquantitative IHC scoring systems for bone tissue studies should simplify and clarify the process of interpretation and reporting of received data.

This review hopefully fulfills the main purpose to present existing approaches to interpretation and presentation of IHC scoring methods and offers researchers assistance with the critical selection and application of scores or appropriate modifications for the individual scientific question. Maybe future investigations even develop new “gold standards” for additional IHC parameters, which would achieve a better comparability between different study results.

## Abbreviations

ALP: Alkaline phosphatase
AR: Antigen retrieval
BMP: Bone morphogenic protein
COL I: Collagen type I
FGF: Fibroblast growth factor
IHC: Immunohistochemistry
IOD: Integrated optical density
IRS: Immunoreactive score
MC: Manual cell counting
MVD: Microvessel density
OCN: Osteocalcin
OD: Optical density
OMP: Objective measurable parameters
OPN: Osteopontoin
OPG: Osteoprotegerin
OTN: Osteonectin
PCNA: Proliferating cell nuclear antigen
ROI: Region of interest
VEGF: Vascular endothelial growth factor
vWF: Von Willebrand factor
